# Silver linings on the horizon: highlights from the 10th Cachexia Conference

**DOI:** 10.1002/jcsm.12290

**Published:** 2018-02-08

**Authors:** Nicole Ebner, Stephan von Haehling

**Affiliations:** ^1^ Department of Cardiology and Pneumology, Innovative Clinical Trials University Medical Center Goettingen Goettingen Germany

**Keywords:** Cachexia, Muscle wasting, Sarcopenia

## Abstract

This article highlights the updates from preclinical and clinical studies into the field of wasting disorders that were presented at the 10th Cachexia Conference held in Rome, Italy, in December 2017. This year's conference saw some interesting results of larger‐scale studies and clinical trials and new therapeutic targets. Herein, we summarize the biological and clinical significance of different markers and new diagnostic tools and cut‐offs for the detection of skeletal muscle wasting, including micro RNAs, the ubiquitin‐proteasome system, mTOR signalling, news in body composition analysis including the D3‐creatine dilution method, and new biomarkers. Clinical studies investigated novel nutritional approaches, trials of elamipretide, enobosarm, and urolithin A. It remains a fact, however, that effective treatments of cachexia and wasting disorders are urgently needed in order to improve patients' quality of life and their survival.

## Introduction

The development of preventive and therapeutic strategies against cachexia and wasting disorders, such sarcopenia, is perceived as an urgent need by health professionals and has instigated intensive research on the pathophysiology of these syndromes.^1^, ^2^, ^3^, ^4^ Cachexia is characterized by progressive weight loss affecting different body compartments, particularly muscle tissue and adipose tissue, although even bone mineral content may be affected.^5^ Over the last years, the Cachexia Conference has developed to a forum for researchers from the fields of cachexia and wasting disorders. It is unique in several ways as it provides a platform for both clinicians and basic researchers to meet and discuss pathways and potential therapeutic targets as well as recent evidence from clinical trials. The fifth conference was held in Rome, and now, the 10th conference was again held in Rome, Italy, from 8 to 10 December 2017 with over 420 participants from more than 25 countries attending and more over 250 posters being presented.

## Basic science

This year, some interesting updates and small molecules on signalling pathways were presented. Especially analysis of genome,^6^ inc RNAs,^7^ mRNAs, proteome,^8^ and microarray were focused this year. So it was of specially interest that Wand *et al*.^9^ (Emory University, Atlanta, GA, USA) presented data of muscle derived exosome miRNA‐26a in chronic kidney disease mice. By using an engineered exosome vector miRNA‐26a generated in muscle satellite cells and injected into the tibialis anterior muscle of chronic kidney disease mice they impressively showed that overexpression of miRNA26a in muscle prevents chronic kidney disease induced muscle loss and cardiac fibrosis via exosome mediated muscle heart crosstalk. It did not come as a surprise that they won the poster price for this presentation. Alexander Ebhardt *et al*.^10^ (Systems Biology Ireland) gave an overview of ageing effects in myogenesis. He showed that highly regulated proteins in foetal are dysregulated in ageing 83 years as function of myogenesis. Also, the energy extraction is lower in ageing, for example, in Krebs cycle. The difference in which signalling cascades are activated seems to be effected by response to tumour necrosis factor α and dysregulation of signalling cascades. He identified the early time points of dysregulated in signalling of interleukin signalling, the activation of the AP‐1 family of transcription factors ERK/ mitogen‐activated protein kinases (MAPK), MAPK targets and MAPK3 (ERK1) activation. In this context, James Carson *et al*. (Center for Colon Cancer Research University of South Carolina) showed that mammalian target of rapamycin (mTOR) signalling is activated after the dark cycle in wild‐type mice and associated with the activation of protein synthesis but can be disassociated from protein synthesis in tumour bearing mice. Overall, there is a suppression of diurnal anabolic signalling in tumour bearing mice, which is related to mTORC1 signalling being disassociated from muscle protein synthesis. Stretch activation of mTOR and protein synthesis is disrupted in by tumour derived media and interleukin‐6 (IL‐6) in cultured myotubes. In tumour bearing mice, mTOR activation is maintained, while the activation of protein synthesis is blocked. Basal and contraction stimulated protein synthesis is differentially regulated by gp130 muscle signalling. The acute activation of mTOR by eccentric contraction is maintained in cachectic muscle but can be disassociated from protein synthesis in tumour bearing mice. One of these important pathways is the ubiquitin‐proteasome system. The ubiquitin proteasome system plays a critical role in skeletal muscle wasting. Studies from many groups over the past years have indeed identified many components in the ubiquitin conjugating system that are induced in atrophying skeletal muscle. This year Didier Attaix *et al*. (Human Nutrition Unit, France) gave an overview to ubiquitination and deubiquitination in skeletal muscle wasting. The ubiquitin specific protease 19 is the deubiquitinating enzyme that has been most studied in muscle wasting. This enzyme is upregulated in muscle in many catabolic conditions and its inactivation leads to protection from muscle loss. Ubiquitin specific protease 19 regulates both protein synthesis and protein degradation as well as myogenesis, thereby modulating the key processes that control muscle mass. Most studies analysed the ubiquitin E3 family and Attaix *et al*. analysed the ubiquitin E2 expression in catabolic C2C12 myotubes (dexamethasone treatment). There seems to be a muscle‐specific muscle RING‐finger protein (MuRF1)‐E2 network with requires stabilization of MuRF1‐E2 complexes by telethonin, a newly identified substrate.^11^ The E2B knockdown induces α‐actin and myosin heavy chain accumulation in the soluble fraction. The isoform E2A is not implicated in the targeting of myofibrillar proteins. MuRF1 interacts with E2E1, E2G1, E2J1, E2J2, and E2L3 and not with E2B, E2D2, E2G2, E2N, and E2V1. This result opens new targets in the field of analysing the pathways of cachexia. Denis Guttridge *et al*.^12^ (Department of Cancer Biology and Genetics, The Ohio State University Wexner Medical Center, Columbus, OH, USA) gave an overview to the important functions of transcription factors nuclear factor κB (NF‐κB) in myoblast to stimulate activin and inhibit muscle differentiation. He impressively showed that NF‐κB is activated in cachectic muscle in both PAX 7 progenitor cells and myofibers.^13^, ^14^ Other studies buttress the view that increased forkhead box protein O (FoxO) signalling and the activation of the transcription factors NF‐κB, MuRF1 and muscle atrophy F‐box (MAFbx) in skeletal muscle play major roles during cachexia onset and progression.^15^, ^16^, ^17^ MuRF1 and MAFbx are essentially involved in muscle atrophy development. Indeed, genes whose expression levels are commonly increased during multiple models of skeletal muscle atrophy, including cancer and sepsis, are MAFbx, MuRF1, and cathepsin, and there is evidence that each are FoxO target genes. Inducers of MuRF1 and MAFbx expression are tumour necrosis factor α, IL‐6 and IL‐1, and NF‐κB appears to be most important regulator of MuRF1 and MAFbx expression in the skeletal muscle.^5^ A number of elegant models were presented in order to improve our understanding of pathways involved in the wasting process. Muscle wasting has received increasing research efforts in recent years.^18^, ^19^ Further research is warranted to investigate the role of decreased physical activity for the suppression of muscle anabolic signalling during the progression of cancer cachexia.^20^ Feeding can activate cachectic muscle mTOR and protein synthesis. Stimulated contractions can attenuate muscle wasting and alter intramuscular cachectic signalling after the initiation of cachexia. Overall, there is a deficit in acute anabolic signalling induced by contraction signalling that is more pronounced than the response to feeding.^21^

## Body composition

Different techniques to measure body composition were presented during the congress including computer tomography (CT) scan, dual energy x‐ray analysis (DEXA) and magnetic resonance imaging, D3‐creatine dilution analysis, and bio impedance analysis. In regards to bio impedance analysis and bioelectrical impedance vector analysis (BIVA) Gonzalez *et al*.^22^ (Rio de Janeiro State University‐Rio de Janeiro, Brazil) showed data from 194 patients with colorectal cancer. They measured skeletal muscle by CT and BIVA. They impressively show that most patients were overweight and phase angle measured with BIVA is associated with body mass index. Moreover they showed that phase angle was independently associated with skeletal muscle mass, muscle function, and muscle quality in colorectal patients. This buttress the view that the measurement of body mass index is not enough.^23^, ^24^ Most studies used CT scan as valid method to detect muscle wasting.^25^ Also Szulc *et al*.^26^ (University of Lyon, France) presented data of high resolution pQCT (xtreme CT Scanco) measured in the STRAMBO study.^27^ They showed the influence of physical performance assessed as score and clinical testing (chair tests, standing with feed in side by side position with eyes open and closed, and 10‐step tandem walk forward and backward). The STRAMBO cohort consists of 817 men aged 60 to 87 years, and physical performance together with grip strength were measured at baseline and after 4 and 8 years. They showed that poor physical performance of the lower limbs is associated with greater decline in distal tibia bone microarchitecture higher risk to fall and higher risk on spine fracture increased high sensitivity c‐reactive protein levels, and poor physical performance is associated with accelerated grip strength decline. One of the main topics this year is the screening tools for the assessment of sarcopenia and frailty and muscle wasting. Steven Heymsfield stated that ‘the challenge is the integrated detection of muscle quality and muscle mass and muscle function’. In regards of screening tools Shafiee *et al*.^28^ (Chronic Diseases Research Center, Endocrinology and Metabolism Population Sciences Institute, Teheran University of Medical Sciences, Teheren, Iran) presented a simple sarcopenia scoring assessment models (SarSAMod) with using data of DEXA, grip strength, and gait speed of 2211 elderly participants. They conclude that this model may can be used as a new tool for detection of sarcopenia. SarSA‐Mod could detect sarcopenia independent of DEXA. Many tools in this regards were presented, for example, the care assessment need (CAN) tool for frailty screening was presented by Priyadarshni *et al*.^29^ (Miami VAHS Geriatric Research Education and Clinic Centre, University of Miami, CA, USA). They compared the CAN score with 40‐item frailty score in 184 participants and concluded that CAN score may offer useful information to primary care for early intervention. Also, the SARC‐F questionnaire was one of the hotline discussions. Beaudart *et al*.^30^ (Research Unit in Public Health, Epidemiology and Health Economics (URSAPES) University of Liége, Liége, Belgium) presented the French translation of the questionnaire. Gonzalez *et al*.^31^ (Rio de Janeiro State University‐Rio de Janeiro, Brazil) showed results of the use of SARC‐F in 205 elderly Brazilian women. They impressively showed that the combination of SARC‐F questionnaire with calf circumference together is the best screening method to rule out healthy subjects from further testing. This would be improving sarcopenia screening in clinical practice.

However, the most amazing method to detect skeletal muscle mass was descripted by Stimpson *et al.,* 32 the D3‐creatine dilution for determination of total body creatine pool size and skeletal muscle mass. This interesting method can directly assess skeletal muscle mass or its change, during ageing, inactivity, disease, or exercise. This method takes advantage of a number of aspects of creatine biology. More than 90% of the total body creatine pool is found in skeletal muscle. Newly synthesized creatine from hepatic and renal sources is transported into the sarcoplasm against a large concentration gradient. 2H labelled creatine is ingested as a 30 mg capsule digested and distributed to skeletal muscle. Creatine is converted to creatinine and excreted in urine. Evans *et al*. (University of California, USA) presented results of a clinical validation study demonstrating that the creatine dilution method is strongly associated with whole body magnetic resonance imaging method.^3^, ^4^ Additionally, they presented that the creatine dilution method is also strongly associated with DEXA method. D3creatine dilution method appears to be a valid non‐invasive measurement for muscle mass and children. In some adults, a portion of the ingested D3creatine label is filtered by the kidney and spilled into urine. By measuring creatine/creatinine ratio, a correction for spilled label is determined with algorithm. Combined with dosing of 2H2O dosing, lean body mass and muscle mass can be measured non‐invasively. Importantly, the measurement with creatine dilution method is not affected by shifts in body water that occur with many cachectic diseases.

## Clinical trials and newly treatment targets

Interestingly, Denis Guttridge *et al*.^12^ (Department of Cancer Biology and Genetics, The Ohio State University Wexner Medical Center, Columbus, OH, USA) presented data from multiplex array to screen a panel of biomarkers of cancer cachexia. With including 50 cachectic patients and 28 noncachectic patients with pancreatic cancer he impressively showed that increases in monocyte chemoattractant protein 1 (MCP‐1) is associated with body weight but not with body mass index. MCP‐1 is a small cytokine that belongs to the chemokine family and recruits monocytes, memory T cells, and dendritic cells to the sites of inflammation produced by either tissue injury or infection. MCP‐1 was found to be higher in cachectic treatment naive pancreatic cancer patients. Additionally, he showed that growth differentiating factor‐15 (GDF‐15) is an important driver of cancer cachexia and transcription factors NF‐κB that is a direct regulator of GDF 15 in pancreatic cancer. GDF15, also known as MIC‐1, is a distant member of the transforming growth factor‐β (TGF‐β) superfamily and has been implicated in various biological functions, including cancer cachexia, renal and heart failure, atherosclerosis, and metabolism better understand the MIC‐1/GDF‐15.^33^ Rangwala *et al*.^34^ (Janssen Research and Development, LLC, Spring House, PA, USA) report that GDNF‐family receptor α‐like (GFRAL), an orphan member of the GFR‐α family, is a high‐affinity receptor for GDF15. GFRAL binds to GDF15 *in vitro* and is required for the metabolic actions of GDF15 with respect to body weight and food intake *in vivo* in mice. They showed that blocking the interaction between GDF15 and GFRAL with a monoclonal antibody prevented the metabolic effects of GDF15 in rats. GFRAL mRNA is highly expressed in the area postrema of mouse, rat, and monkey, in accordance with previous reports implicating this region of the brain in the metabolic actions of GDF15. They concluded that GFRAL represent a new target. In this regards, Breit *et al*.^35^ (St Vincent's Centre for Applied Medical Research, St Vincent's Hospital Sydney, NSW, Australia) presented data from mice model to better understand how prolonged elevation of MIC‐1/GDF‐15 impact animals with adiposity. They showed that mice infused with recombinant MIC‐1/GDF15 (0.5 ug/g body weight/day) for more than 30 days reduces much more fat in obese mice that lean mice and reduces food intake in obese mice. Normal and obese mice respond differently to MIC‐1/GDF15 with a greater weight loss in obese due to loss of fat mass only in normal mice. In obese mice, MIC‐1/GDF15 is highly effective correcting the associated metabolic and inflammatory derangement. The differential effects of MIC‐1/GDF15 are consistent with the relative protection from cachexia afforded by obesity. This year, results of big studies and randomized controlled trials in the field of cachexia, sarcopenia, and muscle wasting were presented. Maria Öhlander *et al*. (CSO smartfish) presented data of the randomized controlled study investigating the effects of Targeted Medical Nutrition (Remune, Smartfish) vs. an isocaloric comparator in chronic obstructive pulmonary disease (COPD) patients.^36^ Remune is high in the omega‐3 fatty acids EPA/DHA and vitamin D and is a source of protein. The product is suitable for patients with precachexia and cachexia who are not able to get sufficient amounts of these nutrients through their normal diet, such as patients with COPD^37^ or cancer. Remune is a milk‐based drink and includes 226 kcal, 10 g protein, omega 3, and 10 μg Vitamin D. A total of 45 patients with moderate to severe COPD with involuntary weight loss receive either Targeted Medical Nutrition (Remune, Smartfish) or isocaloric comparator twice daily for 12 weeks. A significant increase in plasma Vitamin D and omega 3 levels was shown. Only in the remune treated group a significant increase in fat mass and improvement in exercise capacity measured as 6 min walk distance test were shown. A multicompound supplemented nutrition strategy seems to be the right direction.^38^ Interestingly, John Morley stated ‘nutrition is different from malnutrition through both share weight loss’. Roger Fielding *et al*.^39^ (Tufts University, USA) presented the LIFE study a Phase 3 multicentre randomized controlled trial designed to evaluate the efficacy of a long‐term physical activity intervention compared with a successful ageing health education intervention for reducing the incidence of major mobility disability among mobility‐limited older adults. The LIFE study team randomized 1635 participants from eight locations throughout the United States. Daily activity was objectively measured using a hip‐worn, solid‐state triaxial accelerometer (ActiGraph GT3X) at 0, 6, 12, and 24 months according to intervention group that were the participants assigned to attend small group sessions weekly for the first 26 weeks and monthly thereafter. They showed that accelerometry measures could serve as an objective, noninvasive, risk monitoring tool for the older adult population. Moderate‐intensity physical activity programmes significantly improve physical functioning in mobility limited older adults. Combined physical activity and nutritional interventions can positively influence intermuscular fat accumulation and improve muscle quality yet display equivocal effects with respect to physical functioning. Fancesco Landi^40^, ^41^ questioned ‘how much exercise is too much’ Maybe the exercise intervention trial SPRINT‐T study can give an answer. SPRINTT a Phase 3, multicentre, randomized controlled trial aimed at comparing the efficacy in preventing mobility disability of a multicomponent intervention, based on long‐term structured physical activity, personalized nutritional counselling/dietary intervention, vs. a healthy ageing lifestyle education programme. A total of 1519 participants were included until November 2017 when the recruitment was finished, and we are waiting for the first results. Another randomized controlled study MMPOWER presented by Michelangelo Mancuso *et al*. (University of Pisa, Italy) testing Elamipretide also known as bendavia.^42^ MMPOWER is a multicentre, randomized, double‐blind, placebo controlled study included patients between 16–65 years of age with symptoms of mitochondrial myopathy and genetically confirmed mitochondrial disease. Primary mitochondrial myopathies are generally defined disorders leading to defects of oxidative phosphorylation affecting predominantly skeletal muscle. Secondary involvement of mitochondria frequently observed in multiple neuromuscular diseases. The physiological functions of Elamipretide are the restoration of adenosine triphosphate production together with decreased reactive oxygen species emission and electron carriers back together with a higher membrane curvature resulting in normalized cardiolipin content. In the MMPOWER study, 30 patients in Elamipretide dosing groups (*n* = 9 with 0.01 mg/kg/h Elamipretide, *n* = 9 with 0.1 mg/kg/h Elamipretide and *n* = 9 with 0.25 mg/kg/h Elamipretide) compare with 30 patients in placebo group were included. The primary endpoint 6 min walk test at Day 5 was significantly higher in high‐dose group (0.25 mg/kg/h) compared with low dose and placebo. So they started the Phase 3 of the randomized, double‐blind, parallel‐group, placebo‐controlled trial to evaluate the efficacy and safety of daily subcutaneous injections of Elamipretide in subjects with primary mitochondrial myopathy followed by an open‐label treatment extension. We are waiting for these results. Interestingly from literature Elamipretide/Bendavia was also tested in zebrafish lateral cell lines as a novel antioxidant, on gentamicin‐induced hair cell damage.^43^ The treatment of Bendavia exhibited dose‐dependent protection against gentamicin in both acute and chronic exposure. They found that Bendavia at 150 μm conferred optimal protection from either acute or chronic exposure with ototoxin. Bendavia reduced uptake of fluorescent‐tagged gentamicin via mechanoelectrical transduction channels, suggesting its protective effects may be partially due to decreasing ototoxic molecule uptake. The intracellular death pathways inhibition triggered by gentamicin might be also included as no blockage of gentamicin was observed. These data suggest that Bendavia represents a novel otoprotective drug that might provide a therapeutic alternative for patients receiving aminoglycoside treatment.^43^ Also some other new therapeutic molecules were presented this year. For example Serova *et al*.^44^ (Biophytis, UMPC‐BC9, Paris, France) characterize a new small molecule BIO103 *in vitro* and *in vivo* on myocytes in a mouse model. They interestingly showed that using C2C12 cells BIO103 increased myotube diameter consistently with a reduction of Myostatin and atrogin gene expression. BIO 103 activates AKT/mTOR and AMPK signalling that translates into anabolic properties. Other therapeutic treatments include the selective androgen receptor modulators (SARMs). SARMs are a new class of nonsteroidal, tissue specific, anabolic agents that have the potential to increase muscle mass and improve physical function without the unwanted effects on the prostate, skin, or hair that are commonly associated with testosterone or other nonselective, synthetic anabolic steroids. Enobosarm is a nonsteroidal SARM that induces conformational changes in the androgen receptor upon binding, which selectively alters the interaction of the receptor with co‐activator and co‐repressor proteins that exist in different tissues and changes the receptor's ability to regulate gene expression.^45^, ^46^ Improvements in lean body mass and physical function were shown in a Phase 2, double‐blind, placebo‐controlled study of enobosarm in healthy postmenopausal women and elderly men.^5^ This study showed that both 1 and 3 mg of enobosarm resulted in increases in lean body mass in patients with advanced cancer, compared with baseline measurements. This year, a novel selective androgen receptor modulator (TEI‐SARM 2) were presented by Kanou *et al*.^47^ (Teijin Pharma Ltd, Tokyo, Japan). TEI‐SARM2 was administered orally once weekly at 30 mg/kg for 28 days in Duchenne muscle dystrophy rats. They showed that TEI‐SARM2 prevents muscle atrophy. Interestingly, they studied in second analysis the grip force and hindlimb force. They showed that TEI‐SARM2 preserved muscle force in muscle dystrophy rats apart from muscle hypertrophy, so they concluded that TEI‐SARM2 could be a promising drug candidate for various muscle disorders. Most surprisingly in the field of new compounds were presented by Singh *et al*.^48^ (Amazentis SA, Lausanne, Swizerland). They present the new gut metabolite Urolithin A. Urolithin A is a natural gut metabolite produced by host microbiota following transformation of precursors derived from diet. Interestingly, it is not found in food. Urolithin A restores mitochondrial function during ageing (Nature Medicine, 2016). During the mitochondrial life cycle, damaged mitochondria are removed by mitophagy for optimal cell function. Surprisingly, one out of three people have the necessary gut microflora to produce Urolithin A and at variable quantity due to the heterogeneity in gut microflora composition. Urolithin A was tested in 36 elderly participants (mean age 66 years) with nine participants each group (placebo, single intake of 250, 500, and 1000 mg) for 28 days. After a washout phase of 4 weeks, they started multiple dosing for 28 days. Muscle biopsies were taken from each participant. They showed that orally administered Urolithin A is safe when administered in both single and multiple ascending doses to health elderly. They showed a decrease in plasma acylcarnitines with is indicative of improved mitochondrial function. They concluded that Urolithin A impacts biomarkers of mitochondrial function. They impressively showed that oral administered Urolithin A is bioavailable in plasma and skeletal muscle after single dosing and exhibits similar pharmacokinetics with multiple dosing. Genes regulating key mitochondrial pathways are upregulated in human skeletal muscle after 4 weeks oral administration of Urolithin A. Plasma acylcarnitines are lowered with Urolithin A intervention. Long term Phase 2 interventional clinical studies have started in 2017 to investigate the effects of Urolithin A on muscle and mitochondrial function.

## Conclusions

From basic science, new therapeutic targets were shown including the E2‐MuRF1 interaction, gp130 muscle signalling, miRNA‐26a, the interleukin signalling, the activation of the AP‐1 family of transcription factors mitogen‐activated protein kinases, the ubiquitin specific protease 19, MCP‐1 together with GDF 15, and the influence and the role of the gut microbiota in the therapeutic management of muscle wasting and cachexia. Interestingly, the mitochondrial function as target in sarcopenia is newly discussed.^49^ Nevertheless, the definition of cachexia and sarcopenia as well as effective screening tools such as CAN‐score, SarSA‐Mod, and SARC‐F are of special interest. Big randomized controlled studies were presented such as STRAMBO, LIFE, SPRINTT, and MMPOWER. Effective treatments were MIC‐1/GDF‐15, BIO103, enobosarm and TEI SARM2, and gut metabolite Urotilin A.

## Conflict of interest

All authors declare that they have no conflict of interest. The authors certify that they comply with the ethical guidelines for authorship and publishing of the *Journal of Cachexia, Sarcopenia and Muscle*.^50^
